# Alveolar Macrophages in Viral Respiratory Infections: Sentinels and Saboteurs of Lung Defense

**DOI:** 10.3390/ijms26010407

**Published:** 2025-01-05

**Authors:** Pauline Pöpperl, Melanie Stoff, Andreas Beineke

**Affiliations:** 1 Department of Pathology, University of Veterinary Medicine Hannover, 30559 Hannover, Germany; 2 Center for Systems Neuroscience (ZSN), 30559 Hannover, Germany

**Keywords:** alveolar macrophages, tissue-resident macrophages, influenza A virus, innate immunity, respiratory infection, respiratory syncytial virus, SARS-CoV-2

## Abstract

Respiratory viral infections continue to cause pandemic and epidemic outbreaks in humans and animals. Under steady-state conditions, alveolar macrophages (AlvMϕ) fulfill a multitude of tasks in order to maintain tissue homeostasis. Due to their anatomic localization within the deep lung, AlvMϕ are prone to detect and react to inhaled viruses and thus play a role in the early pathogenesis of several respiratory viral infections. Here, detection of viral pathogens causes diverse antiviral and proinflammatory reactions. This fact not only makes them promising research targets, but also suggests them as potential targets for therapeutic and prophylactic approaches. This review aims to give a comprehensive overview of the current knowledge about the role of AlvMϕ in respiratory viral infections of humans and animals.

## 1. Introduction

Global pandemics have made humanity aware of the devastating threat caused by respiratory viral pathogens. The aerogenic route of infection is highly efficient, represented by the highest basic reproduction numbers of infectious diseases, as exemplified in human measles and severe acute respiratory syndrome coronavirus type 2 (SARS-CoV-2) infection [1]. Under the constant threat of new emerging and reemerging viruses, knowledge about host–virus interaction provides the basis for pandemic preparedness. Alveolar macrophages (AlvMϕ) are the most abundant innate immune cells in the deep respiratory tract. Due to their exposed localization on the alveolar luminal surface, they are the first to encounter incoming pathogens and orchestrate the initiation of the immune response in the lung [2,3,4]. A multitude of studies of naturally occurring viral pneumonia and animal models of viral respiratory diseases have been employed to elucidate the relevance of AlvMϕ in the pathogenesis of viral infections. Moreover, omics technology development has given comprehensive insight into molecular mechanisms of viral effects upon AlvMϕ, making them interesting targets for the development of novel therapeutic strategies in infectious disorders [5,6,7]. Targeting AlvMϕ for therapeutic approaches is based on their role as early responders in many infectious diseases, their close proximity to the vasculature, their longevity, and their ability to develop an innate immune memory [7]. The present review aims to summarize the current knowledge about the role of AlvMϕ in viral respiratory diseases in human and veterinary medicine and the effects of AlvMϕ manipulation in experimental models of respiratory virus infections.

## 2. Alveolar Macrophage Biology and Function

AlvMϕ originate from fetal precursors and seed the lung during the embryogenesis phase [8,9,10]. The stable population of AlvMϕ within the lung self-maintains under steady-state conditions [9,11,12,13,14,15,16]. In the healthy lung, AlvMϕ reside approximately in every three alveoli [17]. Due to their localization, AlvMϕ are exposed to both harmless and potentially dangerous particles. This brings the need for a tight regulation with a tolerogenic way of ingesting harmless particles and the induction of proinflammatory responses when needed [18].

**Figure 1:**
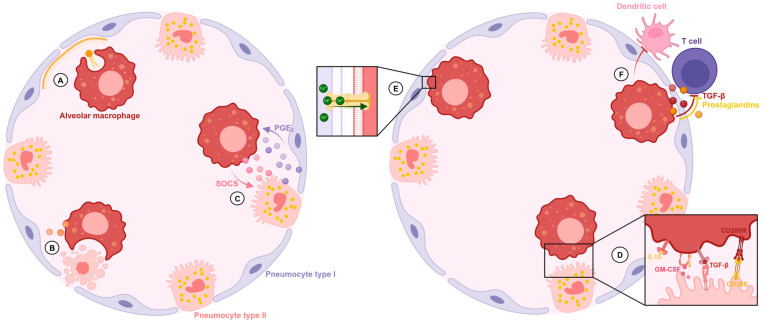
Interaction of alveolar macrophages (AlvMϕ) with their anatomical niche under homeostatic conditions. (**A**). AlvMϕ metabolize and recycle alveolar surfactant. (**B**) AlvMϕ clear apoptotic cells from the alveolar space (efferocytosis) and promote epithelial repair. (**C**) Suppressor of cytokine signaling (SOCS) protein secretion by AlvMϕ influences epithelial responsiveness to cytokines and is enhanced by epithelial-derived Prostaglandin E_2_ (PGE_2_). (**D**) Regulatory interactions between AlvMϕ and epithelial cells secure the tight regulation of AlvMϕ inflammatory reactions via interleukin 10 (IL-10), granulocyte-macrophage colony-stimulating factor (GM-CSF), transforming growth factor-β (TGF-β), and CD200. (**E**) Direct intercellular transduction via gap junction formation between AlvMϕ and epithelial cells and Ca^2+^ waves. (**F**). AlvMϕ mediate suppression of antigen presentation by dendritic cells and T cell proliferation via TGF-β and prostaglandin signaling.

Isolated AlvMϕ adapt to culture conditions, but restore their cell-specific signature following re-transplantation into the lung, reflecting the impact of microenvironment on AlvMϕ phenotype and function [19]. AlvMϕ metabolize and recycle alveolar surfactant, and AlvMϕ depletion has been shown to cause alveolar proteinosis (Figure 1A) [20,21]. Furthermore, pulmonary surfactant per se has a suppressive effect on phagocytosis, which is overcome by toll-like receptor (TLR) 4 signaling [22,23]. The abundance of lipid-rich surfactant and low glucose concentrations in the airways leads to a catabolic lipid metabolism with low levels of glycolysis of AlvMϕ [24]. It has been shown that this distinct metabolism is closely linked to anti-inflammatory and homeostatic properties of AlvMϕ [24,25]. For instance, hypoxia-inducible factor 1-alpha (HIF-1α) expression in AlvMϕ promotes glycolysis-dependent inflammation and lung damage in influenza virus infection [26]. Clearance of apoptotic cells from alveoli (efferocytosis) is another vital function of AlvMϕ (Figure 1B) [2,27,28]. This process inhibits the production of proinflammatory mediators and stimulates the secretion of cytokines, promoting epithelial repair [29,30]. Furthermore, AlvMϕ secrete suppressors of cytokine signaling (SOCS) proteins, which dampen the responsiveness of airway epithelial cells to cytokines [31,32]. Reciprocally, prostaglandin E2 produced by the epithelium enhances SOCS3 expression by AlvMϕ (Figure 1C) [31]. On the other hand, airway epithelial cells secure the tight regulation of AlvMϕ inflammatory reactions by signaling via CD200, transforming growth factor (TGF)-β, CD172a, granulocyte-macrophage colony-stimulating factor (GM-CSF), macrophage colony-stimulating factor (M-CSF), and interleukin (IL)-10 (Figure 1D) [33,34,35,36,37,38]. In addition, communication of AlvMϕ with epithelial cells via gap junctions and Ca^2+^-mediated signal transduction was demonstrated (Figure 1E) [17]. Besides epithelial cells, basophilic granulocytes directly interact with AlvMϕ and regulate their maturation and immunomodulatory function [39]. Interestingly, AlvMϕ are poor antigen presenters and can even suppress antigen presentation by dendritic cells and T cell proliferation by secretion of suppressive prostaglandins and TGF-β (Figure 1F) [40,41,42,43,44,45,46]. Their significance for lung homeostasis is indicated by the fact that AlvMϕ elimination leads to exuberant immune responses to inhaled antigens and airway hyper-responsiveness [47]. AlvMϕ-produced TGF-β also induces regulatory T cells, which prevent Th2-type inflammation in the lung [48,49,50,51]. TGF-β production and low baseline proinflammatory cytokine levels reflect the tolerogenic state of AlvMϕ under steady-state conditions [52].

AlvMϕ are not only influenced by the lung microenvironment, but also by the intestinal microflora. Alterations of gut microbiota and circulating microbial metabolites have been shown to induce memory AlvMϕ and trained immunity in the lung [53].

AlvMϕ are at the ideal location to initiate inflammatory responses to defeat invading pathogens. The switch from the state of tolerance to an inflammatory response is mediated by binding of pathogen-associated molecular patterns (PAMPs) to AlvMϕ pattern recognition receptors (PRRs). Loss of connection to epithelia represent another factor shifting AlvMϕ towards a proinflammatory phenotype [32,54]. TLR activation reduces the responsiveness to anti-inflammatory IL-10 signaling in AlvMϕ [34]. Exceeding the phagocytic capacity of AlvMϕ results in the release of proinflammatory cytokines, including type I interferons (IFNs), tumor necrosis factor (TNF)-α, and IL-1β [55,56,57]. This leads to the recruitment of inflammatory cells and perpetuation of pulmonary inflammation [58]. Subsequently, transcription of interferon-stimulated genes (ISGs) is initiated via Janus kinase/signal transducer and activator of transcription (JAK/STAT) signaling, providing direct antiviral defenses [59,60]. However, inflammatory reactions differ quite markedly depending on the virus, elucidated in more detail in the following chapters. Not only does the activation status and phenotype of AlvMϕ change during virus infection, but also the overall composition of the macrophage pool within the airway niche. AlvMϕ loss in infectious disorders leads to replenishment by innate immune cells. Due to their proliferative capacity, AlvMϕ can partially refill the alveolar niche, but additional recruitment of circulating blood monocytes is necessary under certain pathologic conditions [25,35,56,61,62,63,64,65]. Within the alveolar niche, recruited cells can adapt the distinct phenotype of AlvMϕ [66,67,68,69,70]. However, functional alterations of these recruited AlvMϕ can be either beneficial or detrimental for lung homeostasis, host defense, and tissue repair following respiratory infection [63,64,71,72]. Signals derived from injured epithelial cells promote the repair responses of AlvMϕ with increased growth factor secretion and efferocytosis of apoptotic cells [73,74]. Adaptive effects mediated by epigenetic changes are present in innate immune cells, leading to the development of an innate immune memory (trained innate immunity) [75]. Induction of this state in AlvMϕ has been demonstrated following viral infection, and trained AlvMϕ display a phenotype, which can be described as the “ready to defend” status [76].

Aging is associated with reduced proliferation and responsiveness of AlvMϕ to GM-CSF, accompanied by an increased expression of cellular senescence markers [77,78,79]. Dysregulation of AlvMϕ derived from aged individuals is indicated by increased basal activation levels, decreased IFN-γ responsiveness and increased baseline production of proinflammatory cytokines [77,80]. Moreover, phagocytosis and efferocytosis of apoptotic neutrophils is less efficient in AlvMϕ of aged individuals [81,82,83,84]. The impaired function of AlvMϕ might play a role in the increased risk of the elderly for developing severe pneumonia following respiratory viral infection [85,86].

## 3. Influenza Viruses

Respiratory influenza virus infection has caused multiple pandemic and seasonal epidemic episodes. Especially the elderly, infants, and immunosuppressed people are at risk of developing life-threatening pneumonia, often complicated by secondary bacterial infections. Influenza viruses are enveloped with a segmented, negative-sense single-stranded RNA genome. Most human cases are caused by influenza A virus (IAV, *Alphainfluenzavirus influenzae*) and infection manifests as acute respiratory disease [87,88]. In addition to humans, several mammalian and avian species are susceptible to influenza virus infection, and spillover of highly pathogenic strains from animals to humans occurs [89,90,91]. Classification of IAV is based on their surface protein hemagglutinin (H) and neuraminidase (N).

Host cell entry of IAV involves binding of the H protein to cell-surface sialic acid receptors, followed by endocytosis [92,93]. Permissiveness of human, non-human primate, murine, porcine, canine, and feline AlvMϕ to influenza viruses has been shown [94,95,96,97,98,99,100,101,102,103,104,105,106]. In addition to receptor-mediated virus entry, phagocytosis of virus particles or infected epithelial cells lead to AlvMϕ infection [107]. While in vitro infections of murine and primary human AlvMϕ with H3N2 and H1N1 strains are abortive, productive H5N1 infection of primary human AlvMϕ has been shown [108,109,110,111]. However, systematic screening identified only a subset of H5N1 IAVs able to replicate in human AlvMϕ [112]. For canine H3N8 and swine H1N1 IAV, productive infection of AlvMϕ has been shown in vitro [105,113,114,115,116]. Porcine AlvMϕ cell lines even show productive replication of 11 different IAV strains derived from human, porcine, and avian origin in vitro [117]. In AlvMϕ, several mechanisms of viral inhibition have been recognized, including prevention of viral replication of H1N1 strains and defective viral assembly of seasonal IAV strains [112,118].

Selective depletion and adoptive transfer experiments demonstrate the essential role of AlvMϕ in protection against severe IAV pneumonia. AlvMϕ depletion causes severe symptoms and lung pathology, higher mortality, and enhanced viral replication in animal models. Moreover, AlvMϕ depletion leads to decreased inflammatory cytokine expression in the lung and increased susceptibility to secondary bacterial infections [2,100,119,120,121,122,123,124,125,126,127]. AlvMϕ-derived extracellular vesicles inhibit IAV replication in alveolar epithelial cells in vitro and in vivo, leading to protection of the respiratory epithelium [128]. Thus, an age-related decrease in AlvMϕ counts, accompanied by an impaired ability of AlvMϕ to limit lung damage and compromised efferocytosis of apoptotic neutrophils might account for the increased susceptibility of elderly people to IAV infection [81].

In vivo IAV infection is characterized by early disappearance of AlvMϕ, with repopulation of the alveolar niche within 7–11 days [63,65,101,119,129,130,131]. This initial loss of AlvMϕ is caused by IAV- or host factor-induced cell death [102,119,132]. Pigs infected with avian IAV show an early apoptosis of AlvMϕ, thereby limiting the spread of the virus [102]. Despite their depletion, AlvMϕ contribute to proinflammatory and Antiviral Responses, particularly type I IFN-mediated responses, as shown in mouse models [2]. Interestingly, inhibitory effects of the H1N1 protein NS1 on IL-1β production were shown in porcine AlvMϕ, representing a mechanism of immune evasion [133]. Repopulation of AlvMϕ following early IAV infection is complemented by a massive influx of monocyte-derived macrophages to alveolar spaces [63,65,131]. Early monocyte recruitment is mediated by chemokine (C-C motif) ligand (CCL) 2 and TNF-α released by AlvMϕ [134,135,136]. The expanding macrophage population replaces the original AlvMϕ pool, leading to excessive inflammation, alveolar leakage, and lung pathology, as well as reduced lung defense mechanisms [63,65,127,131,137]. Similarly, loss of AlvMϕ following IAV infection in humans correlates with an increased susceptibility to secondary bacterial infections [119]. As found in IAV mouse infection models, restoration of the original AlvMϕ population is essential for tissue repair following acute lung injury [74].

Detection of IAV by pattern recognition receptors leads to an upregulation of genes related to pathogen sensing, and induction of proinflammatory and antiviral pathways in AlvMϕ [95,110,135,138,139,140,141,142]. RNA sequencing of AlvMϕ from IAV-infected macaques demonstrates a proinflammatory phenotype with prominent chemokine induction [129]. IAV infection of AlvMϕ downregulates the transcription factor peroxisome proliferator-activated receptor gamma (PPAR-γ) via IFN-signaling, which activates proinflammatory pathways [143]. In addition, IAV infection decreases the expression of phagocytosis receptors in human, murine, and turkey AlvMϕ in vitro and in vivo [135,144,145,146,147]. Knockout of the scavenger receptor macrophage receptor with collagenous structure (MARCO) in AlvMϕ improves host survival by heightening chemokine expression during early infection [148,149]. Thus, it seems to be necessary for AlvMϕ to shift from the homeostatic to a proinflammatory phenotype, to induce adequate Antiviral Responses. A second wave of proinflammatory responses is induced after viral clearance, mainly by recruited macrophages in alveolar spaces [150]. While induction of antiviral immune reactions by proinflammatory mediators is vital during early infection, dysregulated cytokine responses and a hyperinflammatory microenvironment cause tissue injury. For instance, IAV-induced IFN-β production by AlvMϕ upregulates pro-apoptotic TNF-related apoptosis-inducing ligand (TRAIL), followed by apoptosis of alveolar epithelial cells in IAV-infected patients [151]. Moreover, TRAIL upregulation in AlvMϕ leads to reduced Na/K-ATPase activity in alveolar epithelial cells, resulting in reduced clearance of edema fluid in the lung, and represents a potential therapeutic target [152].

AlvMϕ-induced T cell activation during IAV infection has been demonstrated, and persistence of IAV nucleoprotein within AlvMϕ maintains lung-resident memory T cells [101,124,153]. Moreover, AlvMϕ attract memory B cells during IAV re-exposure by chemokine expression [154]. Non-neutralizing antibodies promote Fc receptor-mediated endocytosis of viral particles into AlvMϕ, which elicits protective CD8^+^ T Cell Responses in IAV infection [107,155,156,157]. Priming of AlvMϕ with Bacillus Calmette–Guérin vaccine or virus-like particles improves protection against IAV by increasing their efferocytic and antigen presentation capacity [158,159]. GM-CSF has been investigated as a compound to ameliorate IAV disease severity [11,35,36,160,161,162]. Here, intranasal administration or overexpression of GM-CSF prior to IAV infection mediate protection in mice [163,164,165,166,167]. Restoring the homeostatic interaction between CD200 and CD200R also limits weight loss and lung damage in IAV-infected mice [33]. Moreover, intestinal colonization by filamentous bacteria has been shown to enhance AlvMϕ antiviral functions and mediate protection against IAV infection in mouse models [168].

## 4. Coronaviruses

Severe acute respiratory syndrome coronavirus 2 (SARS-CoV-2, *Betacoronavirus pandemicum*) caused a global pandemic from the beginning of 2020 until spring 2023 [169]. Previously, Middle East respiratory syndrome-related coronavirus (MERS-CoV, *Betacoronavirus cameli*) and SARS-CoV (*Betacoronavirus pandemicum*) had caused outbreaks in humans, although these were not as devastating as the recent SARS-CoV-2 pandemic. Coronaviruses are single-stranded, positive-sense RNA viruses with an inner nucleocapsid and an outer membrane containing the spike (S) protein. Coronavirus disease 2019 (COVID-19) usually manifests as an acute respiratory infection [170]. The receptor angiotensin-converting enzyme 2 (ACE2) and cleavage enzymes furin and transmembrane protease serine subtype 2 (TMPRSS2) have been identified as host factors necessary for viral entry, among others [171,172,173,174]. Subsequently, coronavirus S protein cleavage is necessary for host cell infection [173,174]. While expression of MERS-CoV entry receptor dipeptidylpeptidase 4 is present on non-human primate AlvMϕ [175], several studies have focused on the expression of SARS-CoV-2 entrance receptors on AlvMϕ and came to inconsistent results: ACE2, TMPRSS2, furin, and CD147 expression in AlvMϕ have been demonstrated in human lung tissues by immunohistochemistry [176,177]. Contrary to that, studies on primary human AlvMϕ from healthy donors failed to detect ACE2 mRNA in AlvMϕ [178,179,180]. However, the importance of virus entry receptors for AlvMϕ infection has been demonstrated in vitro by ACE2 overexpression, which increases SARS-CoV-2 replication in AlvMϕ of lung explants [181].

Phagocytosis is an alternative cell entry mechanism, leading to SARS-CoV-2 uptake into endosomes and phagosomes, and S protein cleavage by endosomal cathepsin L [173,182,183,184]. S protein cleavage and virus replication are most efficient in AlvMϕ infected with the SARS-CoV-2 Delta-variant [185]. In addition, antibody-bound coronavirus enters AlvMϕ via Fc receptors, as demonstrated in MERS-CoV and SARS-CoV [186,187]. Interestingly, immunization studies in mice suggest that Fc gamma receptor engagement and AlvMϕ are required for vaccine-induced antibody-mediated protection against SARS-CoV-2 infection [188]. Viral protein and RNA have been detected in AlvMϕ of COVID-19 patients [177,178,181,189,190,191,192,193,194,195,196] and permissiveness of AlvMϕ for MERS-CoV and SARS-CoV in murine, common marmoset and rhesus macaques has been demonstrated [175,197,198,199]. Detection of positive- and negative-strand viral RNA of COVID-19 patients provides evidence for active viral replication in AlvMϕ [189]. Similarly, macrophages from bronchoalveolar lavage (BAL) fluids of SARS-CoV-2-infected cynomolgus monkeys contain double-stranded viral RNA, indicative of productive AlvMϕ infection [200].

**Figure 2:**
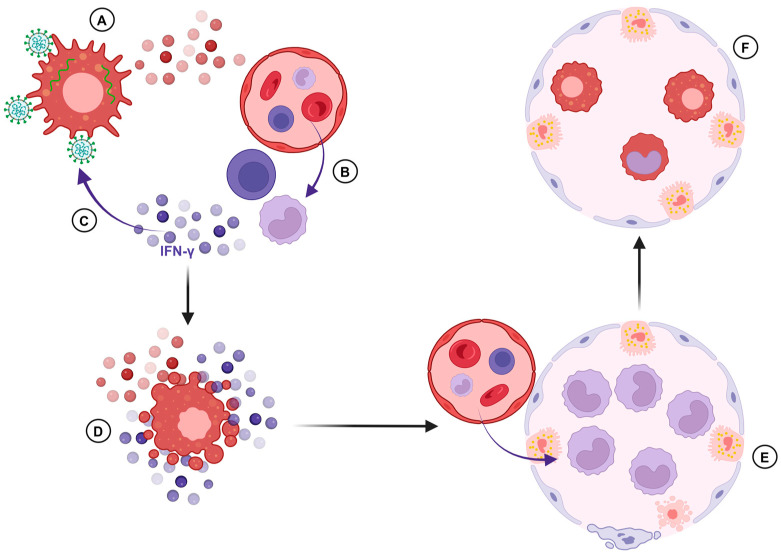
Suggested role of alveolar macrophages (AlvMϕ) in SARS-CoV-2 pathogenesis. (**A**) Activation of AlvMϕ by contact with viral products and subsequent induction of proinflammatory and chemotactic cytokines and chemokines. (**B**) Recruitment of T cells and inflammatory monocytes from the peripheral blood. (**C**) T cell-derived interferon-γ (IFN-γ) further stimulates AlvMϕ activation. (**D**) This hyperinflammatory microenvironment favors dysregulation and death of AlvMϕ. (**E**) Replenishing the lost AlvMϕ; interferon-stimulated, recruited monocytes accumulate within the alveolus and may cause tissue damage. (**F**). In convalescing patients, recruited monocytes differentiate into homeostatic AlvMϕ and alveolar homeostasis is restored.

Depletion of AlvMϕ and replacement by infiltrating immune cells leads to a hyperinflammatory lung environment in severe COVID-19 cases (Figure 2A,B) [201,202,203]. Proinflammatory IL-1β and chemokine expression by AlvMϕ were found in BAL fluid from severely diseased patients [204]. Accordingly, lower inflammatory cytokine expression of AlvMϕ correlates with increased survival [196]. While in mild COVID-19, AlvMϕ upregulate phagocytosis-related pathways and IFN signaling, AlvMϕ from severely diseased patients display increased IL-10 expression, thus inhibiting antiviral IFN responses [205]. Moreover, lung macrophages of patients with severe COVID-19 display reduced expression of genes related to efferocytosis of apoptotic cells [206]. A decrease in tyrosine-protein kinase receptor UFO (AXL) in AlvMϕ of COVID-19 patients is thought to disturb their phagocytic function [207].

AlvMϕ of SARS-CoV-2-infected transgenic mice show a highly activated phenotype [189]. In addition, IFN-signaling by AlvMϕ mediates recruitment of IFN-γ producing T cells, providing a positive feedback loop of macrophage activation (Figure 2C) [189]. The resulting hyperinflammatory microenvironment leads to AlvMϕ cell death and replacement by recruited monocytes (Figure 2D,E). Comparably low numbers of tissue-resident AlvMϕ with loss of their homeostatic properties have been found in BAL fluids of severely diseased COVID-19 patients [189,201,204,207,208]. However, late-phase monocyte differentiation toward a tissue-resident AlvMϕ phenotype with restored homeostatic properties is present in convalescent patients (Figure 2F) [196,204]. Increased AlvMϕ numbers can be found in mild COVID-19 cases, and SARS-CoV-2-infected Syrian golden hamsters with moderate disease severity show no AlvMϕ depletion in the lung [209].

Ex vivo infection of human AlvMϕ with SARS-CoV-2 causes impaired IFN or ISG induction [178]. In this context, the cap structure of coronaviral RNA has been shown to prevent host immune responses via the retinoic acid-inducible gene (RIG)-I/mitochondrial antiviral-signaling protein (MAVS) pathway [210,211]. Experimentally infected cynomolgus macaques show long-term persistence of SARS-CoV-2 in BAL macrophages with reduced production of IFN-γ after lipopolysaccharide stimulation [200]. In vitro M1-polarized murine AlvMϕ take up SARS-CoV-2 more efficiently than M2-polarized cells. The low endosomal pH in M1-polarized AlvMϕ facilitates virus entry and replication [182]. Thus, M1-polarization of AlvMϕ during SARS-CoV-2 infection is supposed to augment viral spread and disease severity [182]. Similarly, AlvMϕ depletion prior to SARS-CoV-2 infection provides protection from lethal disease with enhanced viral clearance and reduced lung pathology in mice [212]. In contrast, AlvMϕ depletion in MERS-CoV-infected mice leads to impaired T Cell Responses with increased lung pathology, symptom severity, and mortality [213]. Epithelial-derived IL-10 upregulates ACE2 and furin expression on murine AlvMϕ ex vivo, increasing their susceptibility to SARS-CoV-2 infection [214].

Several approaches to treating COVID-19 patients focus on AlvMϕ [215]. Based on the hypothesis of lost GM-CSF-mediated differentiation of homeostatic AlvMϕ, inhalation of recombinant human GM-CSF has been shown to boost antiviral immune responses without systemic adverse effects in hypoxic COVID-19 patients [208]. On the other hand, blockage of GM-CSF signaling by intravenous delivery of anti-GM-CSF receptor antibodies can improve the clinical outcome in COVID-19 patients suffering from systemic hyperinflammation [216]. The JAK1/2-inhibitor baricitinib dampens proinflammatory cytokine expression in BAL cells and reduces lung pathology in SARS-CoV-2-infected rhesus macaques [217]. In vitro treatment of human BAL macrophages with IFN-γ reduces viral replication and improves cellular survival [218]. In humanized mice, blocking type I IFN signaling by administering anti-IFN-alpha/beta receptor beta chain (IFNAR2) and the antiviral drug remdesivir was tested [219]. Treatment with a combination of anti-IFNAR2 and remdesivir causes ISG suppression in lung macrophages. Moreover, remdesivir treatment alone leads to reduced levels of proinflammatory cytokines and chemokines in macrophages [219]. Intranasal application of bisphosphate and dexamethasone in SARS-CoV-2 Delta-variant-infected humanized mice downregulates endosomal cathepsin L in AlvMϕ, accompanied by lower viral loads [220]. Another treatment approach regulates the endosomal and lysosomal pH in AlvMϕ via cell-derived microparticles, improving intracellular virus degradation and dampening proinflammatory signaling [183].

## 5. Pneumoviruses

Pneumoviruses are single-stranded, enveloped, negative-sense RNA viruses [221]. Respiratory syncytial virus (RSV, *Orthopneumovirus hominis*) plays a major role as a cause of respiratory tract infections in humans, particularly in premature newborns, infants, and the elderly [222]. RSV has been shown to cause productive infection of AlvMϕ and elicit type I IFN responses, being crucial to limiting viral replication and severity of clinical symptoms [55,223,224,225,226,227,228]. However, as a mechanism of immune interference, RSV and the related bovine RSV (BRSV, *Orthopneumovirus bovis*) in cattle can inhibit IFN-mediated signaling pathways in AlvMϕ [229,230]. RSV infection of murine AlvMϕ triggers the production of proinflammatory mediators, including C-X-C motif chemokine ligand (CXCL) 10, TNF-α, IL-6, CCL3, and CCL4 [231,232,233]. Mouse knockout experiments demonstrate that MAVS and IFNAR signaling are important for the induction of proinflammatory mediators in AlvMϕ upon RSV infection [55,231]. Similar to mice, human AlvMϕ produce TNF-α, IL-6, and IL-8 in response to RSV infection, and BRSV infection of ovine AlvMϕ induces IL-1β, IL-6, IL-8, IL-12, TNF-α, IL-4, and IL-10 expression [234]. Induction of IL-10 in RSV-infected human AlvMϕ might represent an attempt to reduce exuberant proinflammatory responses and maintain alveolar homeostasis [235].

Experimental AlvMϕ depletion prior to RSV infection in mice is associated with decreased production of inflammatory mediators, reduced natural killer cell recruitment, and increased viral titers [224,236,237,238]. Phagocytic clearance of RSV by AlvMϕ has been shown in mouse models [239]. Therapeutic administration of AlvMϕ-like cells generated from pluripotent stem cells decreases lung inflammation and viral loads, indicating a protective function of AlvMϕ in RSV infection [240]. Moreover, AlvMϕ from RSV patients induce adaptive T Cell Response in vitro [226]. However, other studies revealed that AlvMϕ depletion leads to decreased clinical severity, lung inflammation, and viral loads in RSV-infected mice [223,228]. TNF-mediated necroptosis and loss of AlvMϕ disturb viral clearance during early RSV infection [224,228,241,242,243]. Blockage of TNF receptor 1 via intranasally delivered antibodies results in improved clinical disease and elevated macrophage numbers in BAL fluids of infected mice [244].

Patients with RSV infection exhibit an increased susceptibility to bacterial infections of the lung, indicative of disturbed lung defense mechanisms [245,246]. Mouse models reveal a M2-polarization of AlvMϕ following RSV infection, associated with reduced antibacterial function [247]. Moreover, in vitro RSV-infected murine and BRSV-infected bovine AlvMϕ demonstrated a partial loss of their homeostatic function with reduced reactive oxygen species (ROS) production, phagocytosis, and killing of infectious agents [232,248,249]. However, M2-polarization of AlvMϕ might represent a protective mechanism, to prevent immunopathology in RSV infection [250].

RSV reinfection is common in infants, and AlvMϕ might serve as reservoir for viral persistence [251]. Following RSV infection and subsequent reinfection, neonatal mice develop a serious disease characterized by a hyperinflammatory lung environment and decreased amounts of AlvMϕ. Airway macrophage depletion in mice prior to reinfection results in reduced severity of clinical symptoms [252]. The loss of homeostatic AlvMϕ during initial RSV infection is replaced by recruited, potentially harmful macrophages [252]. Compared to adult mice, AlvMϕ from neonates show reduced type I IFN induction and enhanced viral replication upon RSV infection [253]. Furthermore, IL-10 produced by neonatal regulatory B cells negatively impacts the response of neonatal AlvMϕ to RSV infection by reducing their capacity to produce proinflammatory mediators [253,254]. The weak activation of neonatal AlvMϕ coincides with a reduced IFN-γ availability but increased levels of the Th2-cytokines IL-4 and IL-10. Administration of IFN-γ prior to RSV infection improves antigen presentation and proinflammatory cytokine production by AlvMϕ in neonatal mice [243,255].

Regarding AlvMϕ as potential targets for prophylactic and therapeutic approaches against RSV, AlvMϕ depletion of mice prior to vaccination reduces protection upon challenge [256]. Priming of AlvMϕ by probiotics leads to increased production of type I IFNs, IFN-γ, and TNF-α in response to RSV infection, which reduces clinical symptoms, pulmonary inflammation, viral replication, and secondary bacterial pneumonia in mice [257,258,259,260,261,262,263]. Oral administration of *Lacticaseibacillus rhamnosus* improves the antiviral activity of AlvMϕ and leads to enhanced RSV clearance [263]. Another study revealed therapeutic effects of a probiotic mixture on RSV pathogenesis in neonatal mice by increased phagocytosis and IFN-β signaling by AlvMϕ [264]. In addition, intranasal inoculation of the probiotic bacterium *Lactobacillus plantarum* mediates protection of murine pneumonia virus (MPV, *Orthopneumovirus muris*)-infected mice via TLR2 and nucleotide-binding oligomerization domain (NOD) 2 activation [265].

RSV infection is a significant cause of asthma exacerbation in children [266]. Mice recovering from allergic airway eosinophilia show an increased severity of RSV infection, which is mediated by an immature, hyperinflammatory phenotype of AlvMϕ [238]. IL-33 production by AlvMϕ during RSV infection leads to the production of Th2-related cytokines and exacerbation of asthmatic disease [267]. Maturation of AlvMϕ by intratracheal administration of GM-CSF prevents mice from RSV-induced immunopathology, pointing out the significance of AlvMϕ as targets for therapeutic strategies [238].

## 6. Herpesviruses

The *Orthoherpesviridae* family comprises three subfamilies (*Alpha-, Beta-, Gammaherpesvirinae*), and herpesviral disease occurs in a broad range of hosts. Long-term viral persistence is common, and disease develops primarily under immunosuppressive conditions. Herpesviruses are enveloped viruses with a double-stranded, linear DNA genome. In humans, herpes simplex virus 1 and 2 (HSV1/2, *Simplexvirus humanalpha1*), varicella-zoster virus (VZV, *Varicellovirus humanalpha3*), human cytomegalovirus (HCMV, *Cytomegalovirus humanbeta5*), and Epstein–Barr virus (EBV, *Lymphocryptovirus humangamma4*) can cause respiratory disease. Similarly, infectious bovine rhinotracheitis virus (IBRV, *Varicellovirus bovinealpha1*), feline viral rhinotracheitis virus (FVRV, *Varicellovirus felidalpha1*), and equid alphaherpesvirus 1 (EqAHV1, *Varicellovirus equidalpha1*) infections lead to respiratory disease in their respective animal hosts. Ex vivo IBRV infection of bovine AlvMϕ is productive, leading to reduced Fc receptor-mediated phagocytosis [268,269]. In addition, AlvMϕ derived from IBRV-infected calves show reduced production of neutrophil chemoattractants, which increases the susceptibility to secondary bacterial infection [270]. FVRV antigen is present in AlvMϕ of cats with viral pneumonia [271]. Furthermore, macrophage numbers within BAL fluid of EqAHV1-infected ponies are decreased, suggestive of a cytopathic effect on AlvMϕ [272]. In addition, the phagocytic activity of BAL macrophages from infected horses is decreased upon EqAHV1 infection [273]. Pseudorabies virus (PRV, *Varicellovirus suidalpha1*) causes fatal disease in piglets, while adult animals primarily develop respiratory manifestations. PRV infects and replicates in porcine AlvMϕ in vitro and in vivo, with virus strain-dependent cytopathic effects [274,275,276,277]. Depending on the virus strain, infected AlvMϕ also show reduced phagocytosis, phagosome–lysosome fusion, hyperoxide release, and IFN-α production in response to stimulation [278,279]. An enrichment of lipids related to viral assembly and trafficking within PRV-infected AlvMϕ points towards hijacking of the host lipid metabolism for viral replication [277].

Murid gammaherpesvirus 4 (MuGHV4, *Rhadinovirus muridgamma4*) serves as a model for human gammaherpesvirus infection, including EBV, which causes respiratory disease in immunocompromised individuals. AlvMϕ are the first target cells following intranasal infection, showing viral replication and virus transmission to alveolar epithelial cells [64,280,281]. Mice lacking CD4^+^ T cells exhibit higher MuGHV4 infection rates and persistence in AlvMϕ, demonstrating a critical role of CD4^+^ T cell in controlling myeloid cell infection [282]. Futhermore, EBV infection can cause allergic airway disease in human patients [283]. MuGHV4 infection of mice leads to AlvMϕ infection and depletion in alveolar spaces. Subsequent replacement by recruited blood monocytes provides protection against allergic airway disease [64,284]. MuHV4 infection also leads to the polarization of recruited AlvMϕ towards a M2-phenotype, thereby protecting animals against house dust-mite-induced hypersensitivity [284]. Depletion of airway macrophages several weeks after MuGHV4 infection is associated with loss of protection, which confirms the essential role of recruited AlvMϕ [64].

Respiratory HCMV infection is common in humans. Neonatal infection often leads to long-term persistence with frequent periods of reactivation and productive infection. HCMV persists within AlvMϕ in vivo, and productive and lytic infection of primary human AlvMϕ has been shown in vitro [285,286]. Following respiratory murine CMV (MCMV, *Muromegalovirus muridbeta1*) infection of mice and HCMV infection of humans, AlvMϕ are among the first target cells, and infected AlvMϕ spread the infection to other cell types and invade the lung interstitial tissue [281,287,288]. AlvMϕ depletion leads to reduced viral replication in neonatal mice, which demonstrates their role as viral amplifiers [289]. Infection of AlvMϕ depends on MCMV-encoded chemokine 2 (MCK2) expressed by infected cells [289]. Furthermore, lung surfactant increases the uptake of CVM in rat type II alveolar epithelial cells and AlvMϕ [290]. RNA sequencing of infected AlvMϕ reveals a downregulation of antiviral signaling genes related to IFN and nuclear factor kappa-light-chain-enhancer of activated B cells (NF-κB) pathways [287]. Impaired phagocytosis and surfactant clearance are also present in MCMV-infected murine AlvMϕ, leading to increased susceptibility to secondary bacterial infection [287]. However, AlvMϕ depletion prior to MCMV-infection causes higher viral titers, emphasizing antiviral properties of non-infected bystander AlvMϕ [281].

A role for herpesviruses in the pathogenesis of idiopathic pulmonary fibrosis of human patients is suggested, although studies are not conclusive [291,292]. In mouse models of gammaherpesvirus infection, AlvMϕ are partly replaced by recruited monocytes, which display an M2-phenotype and drive the development of pulmonary fibrosis [293]. Other studies demonstrate viral latency in AlvMϕ and an upregulation of profibrotic- and proinflammatory-mediator expression in infected mice [294,295]. Interestingly, gammaherpesvirus equine herpesvirus 5 (EHV5, *Percavirus equidgamma5*) is associated with equine multinodular pulmonary fibrosis [296,297]. Here, EHV5 DNA and intranuclear viral inclusions are present within AlvMϕ of affected horses, although the pathogenesis of fibroses remains undetermined [296,298].

## 7. Retroviruses

Retroviruses are enveloped, single-stranded, positive-sensed RNA viruses with a diploid genome [299]. They are mostly host-adapted and cause a variety of disease syndromes, including immunodeficiency, inflammation, neurodegeneration, and neoplasia. Integration of viral genome components into the host genome is a hallmark of this virus family [300,301]. Human immunodeficiency virus (HIV) 1 and 2 (*Lentivirus humimdef1/2*) cause ongoing public health hazards [302]. Due to improved antiretroviral therapies, disease prevention strategies, and diagnostic tools, HIV infection has become a controllable condition in people with access to health care systems. Initial infection causes flu-like symptoms, followed by a symptom-free period with persistence of HIV provirus DNA in host cells. Acquired immune deficiency syndrome (AIDS) develops in untreated persons and leads to life-threatening opportunistic infections, including respiratory disease [303]. Moreover, lymphocytic alveolitis represents a pulmonary complication in HIV-infected patients, associated with poor prognosis [304,305].

HIV tropism is determined by a combination of the affinity to CD4 receptors and co-receptor usage. Viral entry assays show an inefficient entry and replication of cell-free T cell-tropic HIV, while macrophage (M)-tropic HIV is able to enter and replicate efficiently in human AlvMϕ [306]. Permissiveness of rhesus macaque AlvMϕ to simian immunodeficiency virus (SIV, *Lentivirus simimdef*) has also been demonstrated in vitro and in vivo [307,308,309,310]. Interestingly, T cell-tropic HIV strains can infect AlvMϕ via interaction with infected CD4^+^ T cells and heterotypic cell fusion [306,311]. Close contact of infected AlvMϕ with other cells mediated by intracellular adhesion molecule (ICAM)-1 favors virus spread within AIDS patients [312,313]. In addition, reduced elimination of AlvMϕ by cell death during early SIV infection contributes to viral latency in macaques [314].

People infected with HIV suffer from lung infections, and even patients receiving antiretroviral therapy are at increased risk of developing bacterial pneumonia [315]. A dysregulated lung microenvironment with altered cytokine responses and phenotypic changes in AlvMϕ manifests in HIV patients [309,316,317,318,319,320,321,322,323,324,325]. For instance, increased AlvMϕ-derived TGF-β impairs immunoglobulin secretion in co-cultured peripheral blood mononuclear cells, leading to defective adaptive immune responses [324]. Moreover, AlvMϕ are thought to contribute to lymphocytic alveolitis in HIV patients by releasing chemotactic mediators for cytotoxic CD8^+^ cells [311,326,327,328,329].

Loss of anti-inflammatory surface markers and transcriptomic properties of AlvMϕ from untreated HIV-infected persons have been reported, which coincide with increased TNF-α expression and decreased lung function [330]. Certain miRNAs encoded by HIV stimulate TNF-α production in cultured AlvMϕ [331]. In contrast, other authors reported a reduced ex vivo stimulation-induced TNF-α production and downregulated TLR expression in AlvMϕ from HIV-positive individuals, as well as reduced TNF-α levels in BAL fluid of patients with high viral loads [332]. Downregulation of the transcription factor PPAR-γ in AlvMϕ from HIV patients is thought to cause dysregulated activation profiles [325]. Indicative of disease phase-specific cytokine responses, acute SIV infection causes proinflammatory cytokine secretion by AlvMϕ, which subsequently decreases during chronic infection [317]. Macaques with chronic SIV infection show reduced numbers of AlvMϕ but elevated M1-polarized macrophages in their BAL fluids, leading to a proinflammatory microenvironment in the lung [333]. Microarray analyses reveal an upregulation of several genes associated with classic macrophage activation in AlvMϕ from HIV-positive individuals [323].

Interestingly, HIV preferentially infects a subset of AlvMϕ, representing immature blood monocyte-derived macrophages [334]. Moreover, HIV-infected persons show reduced numbers of homeostatic CD163^+^ AlvMϕ and an increase in alveolar histiocytes of monocytic origin in their BAL fluid [335]. Thus, the proinflammatory lung environment following HIV infection might be caused by recruited monocytes, replacing the tissue-resident AlvMϕ population. Smoking further accelerates the dysregulation of AlvMϕ during HIV infection, leading to enhanced oxidative stress and extracellular matrix remodeling and inflammation, which predisposes the person to chronic obstructive pulmonary disease and pulmonary emphysema [336,337].

Impaired AlvMϕ functions following HIV infection increases the risk of secondary infections. For instance, reduced phagosome activity of AlvMϕ in HIV-infected, untreated individuals is reported [334], and proviral DNA-containing AlvMϕ from HIV patients display reduced phagocytic capacity [338]. AlvMϕ from SIV-infected non-human primates also show impaired antibody-dependent and -independent phagocytosis during the chronic stage of infection [317]. Defective GM-CSF signaling with reduced phagocytic capacity was shown in AlvMϕ of HIV-transgenic rats, related to zinc deficiency [339]. HIV-encoded miR144 inhibits the transcription factor nuclear factor erythroid 2-related factor 2 (NRF2), leading to oxidative stress and impaired phagocytic functions of AlvMϕ in vitro [340,341]. Furthermore, AlvMϕ from seropositive persons show downregulated mannose receptor expression, resulting in reduced binding and phagocytosis of fungi and reduced NF-κB-dependent IL-8 responses in vitro [342]. Defective hydrogen peroxide production by AlvMϕ from asymptomatic HIV-infected persons reduces bactericidal and fungicidal activities of infected AlvMϕ [343]. In addition, AlvMϕ from HIV-infected humans show reduced production of IL-8 and reduced upregulation of inflammatory genes upon bacterial challenge in vitro [323,344]. Conversely, secretion of IL-1β and TNF-α in response to bacterial challenge is increased in AlvMϕ from HIV-positive individuals [323].

AIDS patients are at increased risk of developing tuberculosis. *Mycobacterium tuberculosis* (Mtb) causes a type I IFN-mediated upregulation of Siglec-1, which supports the intracellular spread of HIV in AlvMϕ [345]. Another mechanism of enhanced HIV replication in co-infection with mycobacteria is stimulation of AlvMϕ by activated leukocytes, leading to reduced activity of the inhibitory isoform of CCAAT/enhancer-binding protein beta (C/EBPβ) [346,347,348]. Mtb infection also elevates the expression of HIV co-receptor CXCR4 on AlvMϕ, thereby increasing their permissiveness [349]. Elevated IL-10 levels in BAL fluid of HIV-positive patients inhibit TNF-α responses of AlvMϕ, leading to reduced apoptosis and defective pathogen clearance [350]. Interestingly, antiretroviral treatment had a more negative effect than HIV alone on protective responses to Mtb infection [351].

Although CD4^+^ and CD8^+^ T cells are able to inhibit HIV replication and lyse infected AlvMϕ, persistent infection occurs in the lung [352,353]. The detection of proviral DNA within AlvMϕ in symptomatic and asymptomatic individuals identifies lung innate immune cells as a cellular reservoir for HIV, and AlvMϕ might serve as a source for viral rebound after interruption of antiretroviral therapy [338,354,355,356,357,358,359]. IFN-γ-induced cellular restriction factor adenosine deaminase acting on RNA 1 (ADAR1) acts negatively on HIV replication, thereby favoring persistence of proviral DNA [360]. While viral load in AlvMϕ from asymptomatic patients is very low, it is markedly increased in AIDS patients and secretion of TNF-α, IL-1β, and IL-6 is augmented [318].

Small ruminant lentiviruses include Visna/Maedi virus (VMV, *Lentivirus ovivismae*) in sheep and caprine arthritis encephalitis virus (CAEV, *Lentivirus capartenc*) in goats. Besides encephalomyelitis, arthritis, and mastitis, infections manifest as slowly progressing pneumonia. Although VMV-infected AlvMϕ can be lysed by cytotoxic T cells, a low proportion of AlvMϕ harboring viral protein, proviral DNA, or viral RNA is present in VMV-infected sheep with interstitial pneumonia [361,362,363,364,365,366,367]. Virus infection of AlvMϕ is also shown in CAEV-infected goats [368]. VMV replicates in AlvMϕ in vivo and in vitro [369,370], and infected AlvMϕ are able to transmit VMV across the respiratory epithelial barrier [369]. Following VMV infection, BAL macrophage numbers are elevated and AlvMϕ display an activated phenotype with secretion of IL-8, IL-6, IL-10, GM-CSF, TNF-α, IL-1β, and TGF-β [369,371,372,373,374,375,376]. Furthermore, an increased hydrogen peroxide release of ovine AlvMϕ is found in vitro, likely related to lung hyperreactivity observed in VMV infection [377]. Moreover, cultured VMV-infected AlvMϕ show impaired phagocytosis of bacteria [378].

Feline immunodeficiency virus (FIV, *Lentivirus felimdef*) and equine infectious anemia virus (EIAV, *Lentivirus equinfane*) within AlvMϕ have been shown in infected cats and horses, respectively [379,380]. FIV is an immunosuppressive lentivirus causing AIDS-like disease in cats. However, the role of AlvMϕ in disease pathogenesis has not yet been elucidated. EIAV does not cause productive infection of AlvMϕ in vitro, but increases proinflammatory cytokine expression [381]. Jaagsiekte sheep retrovirus (JSRV, *Betaretrovirus ovijaa*) protein and proviral DNA have been demonstrated in AlvMϕ, but their role in the development of ovine pulmonary adenocarcinoma remains undetermined [382,383].

## 8. Adenoviruses

Adenoviruses are non-enveloped, double-stranded DNA viruses [384]. While in immunocompetent persons infection is usually asymptomatic or manifests with only mild respiratory symptoms, human adenoviruses can lead to severe pneumonia in children and immunocompromised individuals [385]. Human AlvMϕ are permissive to adenovirus infection and account for rapid viral elimination upon infection [386,387]. The scavenger receptor MARCO on AlvMϕ serves as virus entry receptor for human adenoviruses and initiates innate immune responses [388,389]. Knockout mouse models reveal that GM-CSF efficiently increases the phagocytic uptake and lysosomal degradation of adenoviruses by AlvMϕ [390,391]. Internalization of adenoviruses by AlvMϕ also initiates proinflammatory signaling during early respiratory tract infection [392,393]. Here, excessive proinflammatory cytokine release by AlvMϕ is supposed to account for lung injury in adenovirus pneumonia, as shown in experimentally infected hamsters [394,395]. Interestingly, surfactant protein A within alveoli enhances viral clearance by AlvMϕ and inhibits lung inflammation, thereby preventing alveolar injury during pulmonary adenoviral infection of mice [396]. AlvMϕ are the most frequently infected cell type in canine adenovirus pneumonia, which is supposed to interfere with pulmonary defense mechanisms in affected dogs [397,398].

## 9. Morbilliviruses

Morbilliviruses, including human measles virus (MeV, *Morbillivirus hominis*), canine distemper virus (CDV, *Morbillivirus canis*), rinderpest virus (*Morbillivirus pecoris,* eradicated since 2011), peste-des-petits-ruminant virus (PPRV, *Morbillivirus caprinae*), and morbilliviruses of marine mammals cause systemic disease with respiratory distress and profound immunosuppression. Morbilliviruses belong to the family *Paramyxoviridae*, which consists of enveloped, single-stranded RNA viruses [399]. A notable feature of morbilliviruses is their ability to Infect Immune cells of the respiratory tract, from which infectious virus is released before being transmitted to other hosts via aerosols or respiratory droplets. MeV genome can be found in AlvMϕ derived from human BAL fluid [400], and virus antigen has been detected in CD11c^+^ myeloid cells in lungs, representing AlvMϕ and pulmonary dendritic cells, in autopsy and biopsy samples obtained from natural measles cases [401].

**Figure 3:**
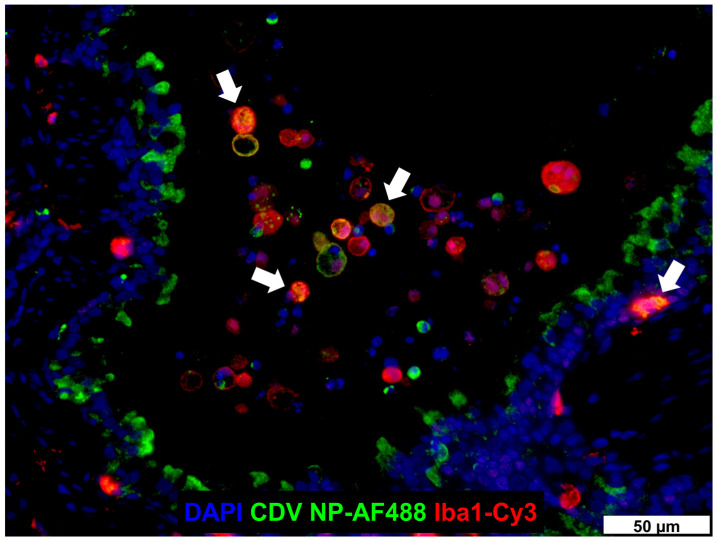
Detection of CDV nucleoprotein (NP) in Iba1^+^ airway histiocytes (white arrows). The photomicrograph by Elisa Chludzinski was published in “Phenotypic and Transcriptional Changes of Pulmonary Immune Responses in Dogs Following Canine Distemper Virus Infection” licensed under CC BY 4.0 (https://creativecommons.org/licenses/by/4.0/, accessed on 1 December 2024) [408].

**Figure 4:**
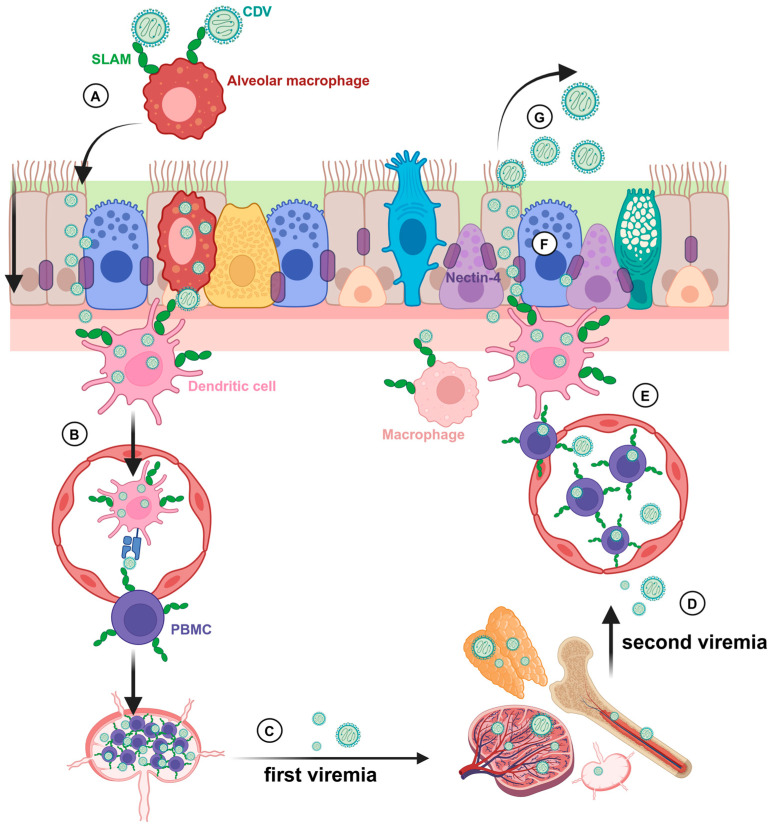
Hypothesized routes of canine distemper virus (CDV) entry and release in the pulmonary system. (**A**) CDV is taken up by SLAM^+^ alveolar macrophages, and bypasses the epithelium or enters epithelial cells directly, via micropinocytosis. (**B**) Professional antigen-presenting cells present CDV antigen to peripheral blood mononuclear cells (PBMCs), in particular lymphocytes. (**C**) CDV replication takes place in regional lymph nodes before dissemination to distant lymphoid tissues. (**D**) During secondary viremia, CDV is transported to the lung. (**E**) CDV-infected immune cells migrate to the basolateral side of the epithelium. (**F**) CDV enters epithelial cells via cellular adhesion molecule nectin-4. (**G**) CDV is released at the apical side to the lumen and propelled rostrally via the mucus layer, ciliary beating, and coughing. Adapted from “Hypothesized routes of CDV entry and release in the pulmonary airway system.” by Elisa Chludzinski, licenced under CC BY 4.0 (https://creativecommons.org/licenses/by/4.0/, accessed on 1 December 2024) [420].

Studies using transgenic mice expressing the viral entry receptor CD150 and experimental infections of non-human primates identified AlvMϕ, besides dendritic cells, as an early target for MeV [402,403,404,405]. Similarly, ferret models show that CDV primarily infects AlvMϕ following aerogenic infection, preceding viral replication in lymphoid organs and viremia [406]. AlvMϕ have been described as the first cell type to become infected with CDV also in canine lung tissue explants [407]. CDV can be detected in airway histiocytes of infected dogs (Figure 3) [408]. It is speculated that MeV and CDV bypass the epithelial barrier of the respiratory tract within AlvMϕ and pulmonary dendritic cells during the early infection phase with subsequent virus dissemination and systemic disease (Figure 4) [407,409].

Virus antigen and viral inclusion bodies in AlvMϕ have been demonstrated in morbillivirus pneumonia of striped dolphins, harbor seals, and harbor porpoises. Like MeV and CDV, the main cytopathic effects of AlvMϕ in these infections include syncytia formation due to distinct viral fusiogenic activity [410,411,412,413]. Moreover, pyknosis of AlvMϕ can be found in horses with pneumonia related to Hendra virus (*Henipavirus hendraense*), another paramyxovirus [414]. As demonstrated by viral histochemistry staining in vitro, feline morbillivirus (*Morbillivirus felis*) exhibits tropism for AlvMϕ, which might contribute to viral entry during early infection. Nevertheless, since the virus is primarily associated with chronic kidney disease in cats, the pathologic relevance of this finding remains uncertain [415]. PPRV also targets AlvMϕ in goats and sheep following aerogenic infection [416,417,418]. Here, in vitro studies revealed that PPRV fusion protein inhibits Antiviral Responses in goat AlvMϕ, leading to increased viral growth and replication. This immune evasion strategy is antagonized by overexpression of plasminogen activator urokinase, enhancing antiviral type I interferon responses in AlvMϕ [419].

## 10. Respiroviruses

The genus *Respirovirus* belongs to the family *Paramyxoviridae,* comprising enveloped, single-stranded, negative-sense RNA viruses, causing acute respiratory disease in humans and animals [399]. Bovine parainfluenza virus 3 (BPIV3, *Respirovirus bovis*) is a component of enzootic pneumonia in calves and bovine respiratory disease complex in feedlot cattle. Like the human parainfluenza virus, BPIV3 infection predisposes to co-infection with other respiratory viruses and bacteria which are related to virus-induced airway lesions and disturbed lung defense mechanisms. BPIV3 replicates within AlvMϕ, and subsequent depression of their phagocytic function and antimicrobial properties has been shown in vitro and in vivo [421,422,423]. Moreover, an increased procoagulant activity of AlvMϕ by BPIV3 is supposed to favor fibrin formation in lung alveoli, leading to a suitable environment for bacterial colonization and growth [424]. Dysfunction of AlvMϕ is associated with an enhanced production of prostaglandins and thromboxane [425]. Thus, targeting the arachidonic acid metabolism of AlvMϕ, particularly reducing prostaglandin E_2_ by cyclooxygenase inhibition, is regarded as a strategy to restore the bactericidal function and pulmonary particle clearance in BPIV3-infected animals [421,426].

Sendai virus (SeV, *Respirovirus muris*) infection of young rodents is used for modeling postinfectious lung disease and virus-triggered asthma. AlvMϕ are thought to play a role in the development of airway injury [427]. For instance, TNF-α released by AlvMϕ contribute to bronchiolar fibrosis in weanling rats upon infection [428]. Moreover, persistence of SeV in lung innate immune cells, including AlvMϕ, may account for the progression from acute to chronic lung disease [429]. With aging, AlvMϕ seem to control asthmatic lung pathology triggered by SeV, since macrophage depletion has been shown to enhance pulmonary type 2 inflammatory responses and exacerbate post-viral lung pathology in experimentally infected adult mice [430].

## 11. Circoviruses

The family *Circoviridae* includes circular, single-stranded DNA viruses [431]. Porcine circovirus 2 (PCV2, *Circovirus porcine2*) is associated with several diseases and syndromes in pigs, known as porcine circovirus-associated diseases (PCV-ADs), contributing to economic losses in the porcine industry [432]. PCV2 infection alone induces only subclinical or mild symptoms, whereas PCV-AD requires additional stimulation mainly by co-infection with other respiratory viruses or bacteria [433,434]. Different organ systems can be affected by PCV-AD, including lymphoid tissues and the lung, where granulomatous lesions develop [432]. Particularly cells of the monocyte/macrophage lineage, including AlvMϕ, are an important target of PCV2, leading to persistent infection [435]. Therefore, AlvMϕ represent a potential virus reservoir, and may facilitate immune evasion and viral spread. Primarily phagocytic or endocytic entry leads to intracytoplasmic location of viral antigen, triggering subsequent cytokine and chemokine responses [436]. Several studies indicate an upregulation of myeloid differentiation primary response (MYD) 88-related signaling pathways upon infection of AlvMϕ in vitro, leading to increased expression of cytokines (e.g., TNF-α, IL-1β, IL-8, IL-10) and chemokines [437,438,439,440]. Imbalanced or prolonged cytokine responses can contribute to impaired pulmonary function, tissue damage, reduced antiviral immunity, and systemic inflammation [440,441]. TNF-α and IL-1β, especially, are associated with the induction of fever, respiratory distress, and lung injury in vivo [439]. Moreover, IL-8, CCL2, CXCL5, and CCL4 are potent chemoattractants for neutrophils, monocytes, and other leukocytes, which induce and maintain granulomatous inflammatory processes [435,437,438,439,440]. Furthermore, PCV2-associated immune modulation of AlvMϕ involves overexpression of IL-10 and TGF-β. Both are potent suppressors of antiviral immunity, promoting viral persistence and facilitating co-infections [442]. The PCV2 capsid protein cap induces IL-10 expression via interaction with the host complement factor receptor gC1qR expressed by AlvMϕ [443]. The same pathway is exploited by PCV2 to suppress IL-12p40 expression, dampening Th1 immune response and further enhancing immunosuppression [444]. In addition to altered cytokine responses, PCV2 also impairs the phagocytic capability of infected AlvMϕ and reduces the production of ROS in vitro, resulting in reduced pathogen clearance and increased susceptibility to secondary infections [434,435].

## 12. Arteriviruses

Arteriviruses are enveloped viruses with a linear, positive-sense RNA genome [445]. Porcine reproductive and respiratory syndrome virus (PRRSV-1/2, *Betaarterivirus europensis/americense*) accounts for severe economic losses in the swine industry by causing respiratory disease primarily in young pigs and reproductive failure in sows. Respiratory infection results in interstitial pneumonia and impaired pulmonary innate immunity, facilitating secondary bacterial and other viral infections [446]. AlvMϕ are primary target cells for initial PRRSV replication during acute infection, which precedes virus spread, while persistent infection of these cells causes chronic respiratory problems in affected pigs [447]. PRRSV leads to reduced phagocytic capacity and bactericidal function of AlvMϕ, as shown in vitro and in vivo [448,449,450]. Moreover, PRRSV elicits only weak antiviral innate immune responses in AlvMϕ, mediated by virus protein interference with molecules of the type I IFN pathway. Here, PRRSV non-structural protein 1 (nsp1) is considered as the strongest antagonist of IFN-β production, by acting on the phosphorylation and nuclear translocation of interferon regulating factor 3 [451,452]. In addition, PRRSV induces a M2-phenotype of AlvMϕ, characterized by CD163 expression, which serves as a virus entry receptor, while simultaneously counteracting Antiviral Responses of M1-polarized macrophages [453,454,455].

Impaired antigen processing and major histocompatibility complex class I-restricted antigen presentation lead to reduced cytotoxic T Cell Responses, following infection with highly pathogenic PRRSV strains [456]. In vitro studies also demonstrate reduced cytotoxic function of natural killer cells against PRRSV-infected AlvMϕ [457]. Transcriptome analyses of PRRSV-infected AlvMϕ reveal an upregulation of genes related to T cell exhaustion, including indoleamine-2,3-dioxygenase and the immune check point molecules programmed cell death-ligand (PDL)-1 and PDL-2 [458,459]. Moreover, increased expression of suppressive cytokines IL-10 and TGF-β by AlvMϕ following PRRSV infection are supposed to impair T cell function. These mechanisms contribute to adaptive immune evasion and chronic respiratory infection in pigs [460,461].

PRRSV transiently impairs the expression of proinflammatory cytokines in the early stage of infection. However, the virus is able to induce an inflammatory storm during the late stage [462]. In particular, highly pathogenic PRRSV elicits excessive secretion of proinflammatory cytokines, such as TNF-α, IL-8 and IL-1β, thought to foster diffuse alveolar damage and pulmonary edema in infected pigs. Although less efficient than pulmonary intravascular macrophages, infected AlvMϕ participate in endothelial barrier injury by releasing inflammatory mediators, as demonstrated in vitro using transwell co-culture systems [463,464,465].

A time-dependent increase in AlvMϕ apoptosis has been found in pigs experimentally infected with PRRSV [466]. In vitro experiments demonstrate an activation of anti-apoptotic pathways during initial infection, thereby favoring viral replication. Late in infection, PRRSV-infected AlvMϕ die by apoptosis and secondary necrosis [467]. In addition to apoptosis, highly pathogenic PRRSV induces pyroptosis of AlvMϕ in vitro and in vivo, associated with excessive IL-1β secretion. Triggering programmed cell death in infected AlvMϕ is regarded as a mechanism to restrict viral replication and promote PRRSV elimination, but might also contribute to immunopathology and lung damage in affected animals [468]. Given the central role of AlvMϕ in PRRSV infection and pathogenesis, the development of therapeutics strengthening Antiviral Responses and maintaining homeostatic functions of these cells represents a novel approach in porcine health management [469,470].

Equine arteritis virus (EAV, *Alphaarterivirus equid*) is the causative agent of equine viral arteritis. The course of disease can range from asymptomatic cases to respiratory and reproductive symptoms, depending on virus strain virulence, route of transmission, and host immunity [471]. Horizontal transmission occurs during the acute disease phase via aerosols, and, like PRRSV, AlvMϕ represent the primary cells for viral entry. EAV antigen can be demonstrated within AlvMϕ of infected horses and aborted fetuses [472]. Similar to cytokine expression of PRRSV-infected porcine AlvMϕ, EAV infection of equine AlvMϕ induces an increased expression of proinflammatory cytokines such as IL-1β, IL-6, IL-8, and TNF-α. The majority of clinical signs in equine viral arteritis are related to vascular injury [381]. In vitro studies have shown that EAV-infected AlvMϕ activate endothelial cells, resulting in increased expression of adhesion molecule E-selectin, and potentially release vasculotoxic IL-1β and TNF-α [381].

Several factors impair the efficacy of vaccination, and insufficient protection is observed particularly in multifactorial diseases [473]. Therefore, antiviral therapeutics that act directly on local target cells, such as AlvMϕ, have been explored. For instance, matrine, a plant-based alkaloid, is able to inhibit the replication of PRRSV and PCV2 in AlvMϕ in vitro, probably by interfering with TLR signaling, NF-κB-related pathways and virus-induced TNF-α release [474].

## 13. Conclusions

The reported studies delineate a multifaceted function of AlvMϕ in viral respiratory diseases. Animal models and in vitro experiments reveal beneficial and detrimental effects of the cells during infection, which clearly depend on virus properties, disease phase, intervention strategies, and the age of the host. AlvMϕ promote virus amplification and spread during early infection, and can serve as a reservoir for persistent infection. Since proper function of AlvMϕ are critical for lung defense mechanisms and viral elimination, several pathogens have evolved efficient strategies to evade host immune responses, particularly by interfering with type I interferon signaling. In addition, viral hijacking of the cellular machinery has the ability to impair pathogen recognition and the phagocytic capacity of AlvMϕ, increasing the host’s susceptibility to pulmonary co-infections [2,3,4]. Moreover, dysregulated cytokine responses and loss of homeostatic function of AlvMϕ during virus infection can enhance airway hyper-reactivity and virus-induced immunopathology. Given their plasticity and different functions in respiratory diseases, therapeutic strategies targeting AlvMϕ should be based on the needed role in a given disease situation. Although many strategies mentioned in this review article give hope to improving human and animal health in the future, more work is clearly needed to transfer in vitro and animal experimental results to clinical settings. While their position in the deep lung bears great potential for efficient therapeutic strategies, it also brings challenges, such as avoiding overloading of AlvMϕ, side effects in other cell populations, or compound loss in the upper airways [7]. Therefore, research on efficient and safe drug-delivery systems is needed to realize AlvMϕ-targeted therapeutic approaches. Understanding the unique properties of AlvMϕ and their complex interplay with other cells of the lung immune system represents a prerequisite for the development of new therapeutic approaches in respiratory virus infections.

## Data Availability

Not applicable.
